# An Improved and Secure Anonymous Biometric-Based User Authentication with Key Agreement Scheme for the Integrated EPR Information System

**DOI:** 10.1371/journal.pone.0169414

**Published:** 2017-01-03

**Authors:** Jaewook Jung, Dongwoo Kang, Donghoon Lee, Dongho Won

**Affiliations:** Department of Computer Engineering, Sungkyunkwan University, 2066 Seoburo, Suwon, Gyeonggido 16419, Korea; King Saud University, SAUDI ARABIA

## Abstract

Nowadays, many hospitals and medical institutes employ an authentication protocol within electronic patient records (EPR) services in order to provide protected electronic transactions in e-medicine systems. In order to establish efficient and robust health care services, numerous studies have been carried out on authentication protocols. Recently, Li et al. proposed a user authenticated key agreement scheme according to EPR information systems, arguing that their scheme is able to resist various types of attacks and preserve diverse security properties. However, this scheme possesses critical vulnerabilities. First, the scheme cannot prevent off-line password guessing attacks and server spoofing attack, and cannot preserve user identity. Second, there is no password verification process with the failure to identify the correct password at the beginning of the login phase. Third, the mechanism of password change is incompetent, in that it induces inefficient communication in communicating with the server to change a user password. Therefore, we suggest an upgraded version of the user authenticated key agreement scheme that provides enhanced security. Our security and performance analysis shows that compared to other related schemes, our scheme not only improves the security level, but also ensures efficiency.

## Introduction

**Fig 1:**
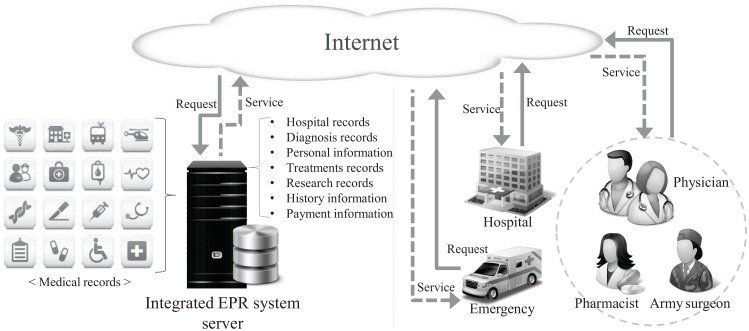
Integrated EPR information system.

The development of Information and Communication Technology (ICT) with the prevalent use of the mobile Internet, smart devices, social network services, and cloud services has brought remarkable changes to our daily lives. This development has also affected the medical field, which has retained a number of conventional and inefficient methods. Recently, a large number of hospitals providing health care services have instituted EPR systems in order to remotely communicate with patients, and to efficiently process their medical records and disease management [1]. The EPR system allows the sharing of patients’ medical histories, such as hospital records, diagnosis records, personal information, treatment records, and research records. Using the EPR system, all patient information is available electronically, on screen, at any hospital location, at any time. In addition, EPR provides the most recent and accurate information, enabling faster diagnoses, treatment plans and discharge processes for the patient [2]. Fig 1 illustrates the integrated EPR information system.

While the users enjoy simplicity and efficiency in EPR information systems, security has emerged as a major issue in both academic and industrial fields [3, 4]. In order to guarantee reliability, authentication protocol provides security and mutual authentication when users access a foreign network.

Lamport [5] first presented an authentication technique based on passwords, and since then, many related studies [6–22] have been conducted to improve security and efficiency for various environments such as wireless environments, Telecare medical information systems (TMIS), health care systems, wireless sensor networks (WSNs), multi-server environments, and mobile pay-TV systems. In 2006, Lee et al. [6] presented a security enhanced authentication mechanism for wireless environments. Wu et al. [7], He et al. [8], Hao et al. [9], Jiang et al. [10] and Moon et al. [11] have proposed authentication method for TMIS. Amin et al. [12] and He et al. [13] proposed user anonymous authentication scheme for health care systems. In 2014, Kim et al. [14], Choi et al. [15] and Nam et al. [16] proposed authenticated key agreement mechanisms for WSN environments. In the same year, Khan [17] presented a fingerprint biometric-based self-authentication and deniable authentication scheme for electronic transaction. In addition, Chaudhry et al. [18], Amin & Biswas [19], Moon et al. [20], and Mishra et al. [21] presented authentication systems for multi-server environments. In 2016, He et al. [22] presented one-to-many authentication using bilinear pairing in mobile pay-TV systems.

Especially, in order to improve the security, various cryptography techniques are used in authentication protocol. Khan et al. [23] and Lee & Hsu [24] apply a chaotic map technique in their scheme. In 2015, Giri et al. [25] presented an RSA-based authentication method for TMIS. However, Amin & Biswas [26] demonstrated that Giri et al.’s scheme [25] cannot guarantee protection against off-line password guessing attacks and insider attacks, and suggested an improved mechanism based on an RSA cryptosystem. In addition, Chaudhry et al. [27], Irshad et al. [28], Islam & Khan [29], Amin & Biswas [30], and Amin et al. [31, 32] presented authentication mechanisms using elliptic curves cryptography (ECC) for TMIS.

In 2012, Wu et al. [33] first presented an efficient user authentication technique for an integrated EPR information system. They used lightweight operations in their protocol including one-way hash operations and bitwise XOR operations in order to enhance efficiency. However, Lee et al. [34] pointed out that Wu et al. [33] overlooked the possibility of stolen smart card attack through power consumption analysis [35]. Lee et al. [34] then suggested an improved version that addressed the issue of Wu et al.’s [33] technique. However, Wen [36] demonstrated that Lee et al.’s scheme [34] still had some weaknesses, such as off-line password guessing attacks and user impersonation attacks, and proposed an enhanced new strategy. Unfortunately, Li et al. [37] demonstrated that Wen’s scheme [36] cannot prevent password disclosure attack nor provide efficient password change. In 2015, Das [38] discovered that Lee et al.’s scheme [34] and Wen’s scheme [36] shared the same three vulnerabilities. First, the password change phase of both schemes had no verification process of the user’s previous password. Second, their schemes were not protected against insider attack. Third, in their studies, formal security analysis was not conducted. In an attempt to compensate for these defects, Das [38] presented an upgraded scheme. However, Mir et al. [39] discovered that Das’s scheme [38] is not protected against off-line/on-line password guessing attacks, and propose a secure anonymous authentication mechanism. Li et al. [40] recently also demonstrated that Das’s scheme [38] could not satisfy security requirements because it is not protected against modification attacks and user duplication attacks. They then suggested an enhanced new authentication mechanism.

However, we have discovered that Li et al.’s scheme [40] comprises critical security weaknesses. Their scheme: (i) cannot prevent off-line password guessing attacks and server spoofing attacks, (ii) is unable to preserve user anonymity, (iii) does not identify incorrect passwords promptly in login stage, and (iv) has non-user-friendly password changing procedure, since it requires communication with the server. In this current research, our main contribution is as follows. First, we describe the weaknesses of the above scheme. Second, we propose a more developed authentication mechanism for an integrated EPR information system. Third, we show that the proposed mechanism satisfies the various security requirements. Finally, we demonstrate that the proposed mechanism has good performance in terms of computation cost and time consumption.

The remainder of this paper is structured as follows. Section 2 provides some background information on prior knowledge. In Section 3, we briefly explain Li et al.’s authentication procedure. Section 4 demonstrates the vulnerabilities of Li et al.’s scheme. A detailed explanation of our proposed scheme is provided in Section 5. In Section 6, we evaluate whether our proposed scheme can withstand various attacks while satisfying our claim that the basic requirements of the security scheme are provided. In Section 7, we analyze the performance of the proposed scheme and in Section 8, we provide a conclusion to the paper.

## Preliminary Knowledge

In this section, we will describe basic knowledge in terms of security properties and introduce bio-hash function [41], which is used in our proposed scheme.

### Security requirements

Multiple security requirements should be considered in order to implement a secure and efficient authentication mechanism. In this subsection, based on previous researches [33, 34, 36, 38, 40, 42–44], we outline some of the important requirements of an authentication scheme. In Section 6, these requirements will be employed in order to scrutinize the security of prior schemes and our proposed scheme.

1. **User anonymity**: In an authentication mechanism, even if an attacker extracts some information stored in a smart card or eavesdrops the exchanged message in the communication group, the user’s identity should be preserved.
2. **Mutual authentication**: An authentication mechanism should execute several steps to achieve mutual authentication which is to test all transmitted messages to judging the legitimacies.
3. **Session key agreement**: After the verification process has completed, the user and server should assign the session key to each other.
4. **Password verification process**: If a user erroneously enters an incorrect password in the login phase, the password should be detected before performing the verification phase.
5. **User friendliness**: An authentication mechanism provides a password change procedure with which a user can freely update their password without communicating with the server.
6. **Robustness**: An authenticated key agreement mechanism should be immune to different types of attacks, such as insider attacks, off-line password guessing attacks, replay attacks, and user impersonation attacks.

### Bio-hash function

Recently, three-factor authentication mechanism has frequently been used, which complements the two-factor authentication mechanism using *ID* and *PW* by adding biometric information in order to increase security. In a number of studies on the three-factor authentication mechanism [11, 20, 21, 45, 46], the bio-hash function has been applied to the user’s biometric information. In 2004, Jin et al. [41] suggested a fingerprint-based function to identify the user’s legitimacy. The bio-hash technique employs the particular tokenized pseudo-random numbers to each of users measuring biometric feature arbitrarily onto two fold strands. The bio-hash function *H*(⋅) is a one-way function with a feature that can reduce the probability of denial of service. In order to improve security, our proposed scheme adopts the user’s biometric information applied in the bio-hash function. The details are as follows in Section 5.

## Description of Li et al.’s scheme

**Fig 2:**
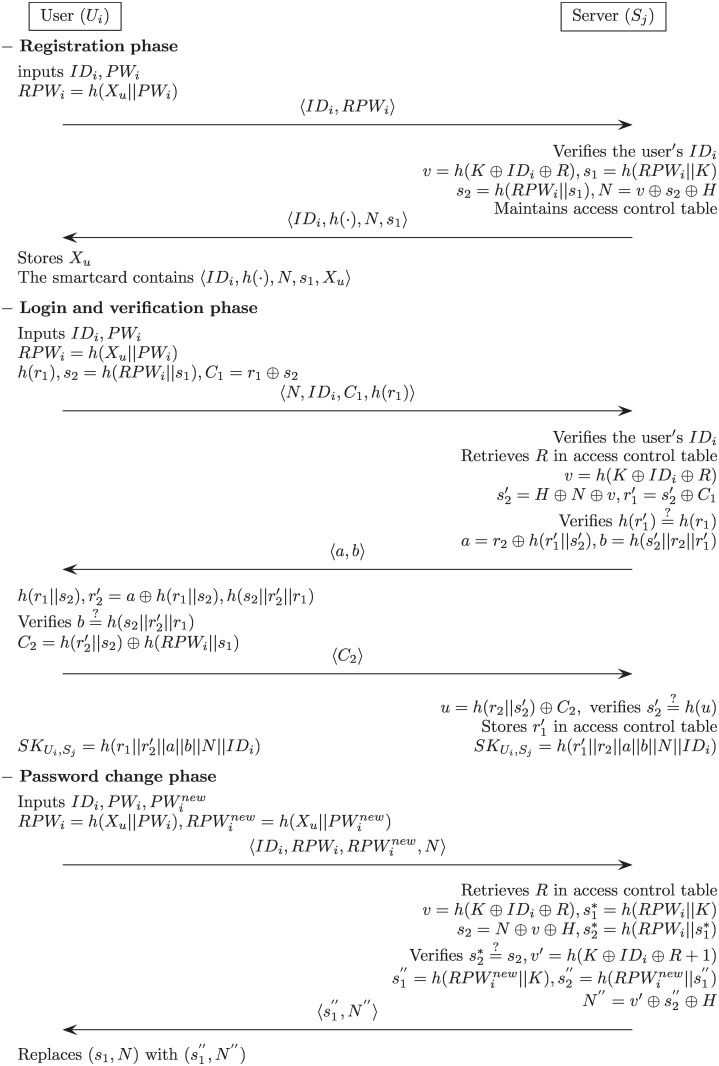
Li et al.’s authentication scheme.

In this section, we briefly review Li et al.’s authentication mechanism [40] in order to cryptanalyze their scheme. Their scheme consists of the following phases: registration, login, verification, and password change. Fig 2 describes Li et al.’s scheme, and Table 1 shows the notations employed in the remainder of this paper.

**Table 1:** Notations.

Notations	Description
*U*_*i*_	User
*S*_*j*_	EPR information system server
*ID*_*i*_	Identity of *U*_*i*_
*PW*_*i*_	Password of *U*_*i*_
$PW_{i}^{new}$	New password of *U*_*i*_
*R*	Number of times *U*_*i*_ re-registers to *S*_*j*_
*B*_*i*_	Biometric information of *U*_*i*_
*X*_*u*_	Secret number generated by *U*_*i*_
*H*	Secret number generated by *S*_*j*_
*K*	Secret key of *S*_*j*_
*r*_1_	Random number generated by *U*_*i*_
*r*_2_	Random number generated by *S*_*j*_
*h*(⋅)	One-way hash function
*H*(⋅)	Bio-hash function
*X*||*Y*	Concatenate operation
⊕	XOR operation

### Registration phase

1. *U*_*i*_ inputs his/her *ID*_*i*_ and *PW*_*i*_, and *U*_*i*_ generates a random secret number *X*_*u*_ that is only retained by user *U*_*i*_. *U*_*i*_ computes *RPW*_*i*_ = *h*(*X*_*u*_||*PW*_*i*_) and sends a registration request message 〈*ID*_*i*_, *RPW*_*i*_〉 to *S*_*j*_ through a secure channel.
2. *S*_*j*_ verifies the user’s *ID*_*i*_. If it is valid, *S*_*j*_ computes *v* = *h*(*K* ⊕ *ID*_*i*_ ⊕ *R*), where the secret number *K* is chosen by *S*_*j*_.
3. *S*_*j*_ computes *s*_1_ = *h*(*RPW*_*i*_||*K*), *s*_2_ = *h*(*RPW*_*i*_||*s*_1_) and *N* = *v* ⊕ *s*_2_ ⊕ *H*. *S*_*j*_ then issues a smart card with the parameters {*ID*_*i*_, *h*(⋅), *N*, *s*_1_} and sends it to *U*_*i*_ through a secure channel. At this time, *S*_*j*_ constructs an access control table. This table includes the user’s identity *ID*_*i*_ in the first field and in the case of initial registration of *U*_*i*_, records *null* value and *R* = 0 into the second and third fields, respectively. If it is not an initial registration, *S*_*j*_ records *R* = *R*+1 in the existing field.
4. Upon receiving the smart card, *U*_*i*_ enters the secret number *X*_*u*_ into its memory, and the smart card includes the information {*X*_*u*_, *ID*_*i*_, *h*(⋅), *N*, *s*_1_}.

### Login phase

1. *U*_*i*_ inserts *U*_*i*_’s smart card into a card reader and inputs his/her *ID*_*i*_ and *PW*_*i*_. The smart card then computes *RPW*_*i*_ = *h*(*X*_*u*_||*PW*_*i*_).
2. The smart card selects a random number *r*_1_ and computes *h*(*r*_1_), *s*_2_ = *h*(*RPW*_*i*_||*s*_1_) and *C*_1_ = *r*_1_ ⊕ *s*_2_.
3. Finally, *U*_*i*_ sends the login request message 〈*N*, *ID*_*i*_, *C*_1_, *h*(*r*_1_)〉 to *S*_*j*_ through a public network.

### Verification phase

1. *S*_*j*_ verifies the user’s *ID*_*i*_. If it holds, *S*_*j*_ accepts the login request and proceeds with the next step. Otherwise, *S*_*j*_ rejects the login request and this phase is terminated.
2. *S*_*j*_ retrieves the *R* stored in the access control table and computes *v* = *h*(*K* ⊕ *ID*_*i*_ ⊕ *R*), $s_{2}^{\prime }=H⊕N⊕v$ and $r_{1}^{\prime }=s_{2}^{\prime }⊕C_{1}$. *S*_*j*_ then verifies whether $h\left(r_{1}^{\prime }\right)=h\left(r_{1}\right)$. If this holds, *S*_*j*_ proceeds to the next step. Otherwise, this phase is terminated.
3. *S*_*j*_ selects a random number *r*_2_ and computes $a=r_{2}⊕h(r_{1}^{\prime }\left|\right|s_{2}^{\prime })$ and $b=h(s_{2}^{\prime }||r_{2}||r_{1}^{\prime })$. *S*_*j*_ then sends an authentication request message 〈*a*, *b*〉 to *U*_*i*_ through a public network.
4. *U*_*i*_ computes *h*(*r*_1_||*s*_2_), $r_{2}^{\prime }=a⊕h(r_{1}\left|\right|s_{2})$ and $h(s_{2}||r_{2}^{\prime }||r_{1})$. *U*_*i*_ then verifies whether $b=h(s_{2}||r_{2}^{\prime }||r_{1})$. If this holds, *U*_*i*_ successfully authenticates *S*_*j*_. Subsequently, *U*_*i*_ computes $C_{2}=h(r_{2}^{\prime }\left|\right|s_{2})⊕h(RPW_{i}\left|\right|s_{1})$ and sends the acknowledgement message 〈*C*_2_〉 to *S*_*j*_ through a public network.
5. *S*_*j*_ computes $u=h(r_{2}\left|\right|s_{2}^{\prime })⊕C_{2}$ and verifies whether $s_{2}^{\prime }=h\left(u\right)$. If this holds, *S*_*j*_ successfully authenticates the *U*_*i*_ and stores $r_{1}^{\prime }$ in its access control table.
6. Finally, *S*_*j*_ computes a shared session key $SK_{U_{i},S_{j}}=h(r_{1}^{\prime }\left|\right|r_{2}||a||b||N||ID_{i})$ and the *U*_*i*_ also computes the same shared session key $SK_{U_{i},S_{j}}=h(r_{1}\left|\right|r_{2}^{\prime }||a||b||N||ID_{i})$ successfully.

### Password change phase

1. *U*_*i*_ inserts *U*_*i*_’s smart card into a card reader and inputs *ID*_*i*_, old password *PW*_*i*_ and new password $PW_{i}^{new}$. The smart card computes the old masked password *RPW*_*i*_ = *h*(*X*_*u*_||*PW*_*i*_) and the new masked password $RPW_{i}^{new}=h(X_{u}||PW_{i}^{new})$. *U*_*i*_ then sends the password change request message $\langle ID_{i},RPW_{i},RPW_{i}^{new},N\rangle$ to *S*_*j*_ through a secure channel.
2. *S*_*j*_ retrieves *R* stored in the access control table and computes *v* = *h*(*K* ⊕ *ID*_*i*_ ⊕ *R*), $s_{1}^{*}=h(RPW_{i}\left||K\right)$, *s*_2_ = *N* ⊕ *v* ⊕ *H*, and $s_{2}^{*}=h(RPW_{i}\left|\right|s_{1}^{*})$. *S*_*j*_ then verifies whether $s_{2}^{*}=s_{2}$. If this holds, *S*_*j*_ accepts the password change request and proceeds with the next step. Otherwise, the password change request is rejected and this phase is terminated.
3. *S*_*j*_ computes *v*′ = *h*(*K* ⊕ *ID*_*i*_ ⊕ *R*+1), $s_{1}^{\prime \prime }=h(RPW_{i}^{new}\left||K\right)$, $s_{2}^{\prime \prime }=h(RPW_{i}^{new}\left|\right|s_{1}^{\prime \prime })$ and $N^{\prime \prime }=v^{\prime }⊕s_{2}^{\prime \prime }⊕H$. *S*_*j*_ then sends the password change update message $\langle s_{1}^{\prime \prime },N^{\prime \prime }\rangle$ to *U*_*i*_ through a secure channel.
4. Upon receiving the password change update message, the smart card replaces the existing values *s*_1_ and *N* with the new values $s_{1}^{\prime \prime }$ and *N*′′, respectively. Finally, the smart card contains the information $\left\{ID_{i},h\left(\cdot \right),N^{\prime \prime },s_{1}^{\prime \prime },X_{u}\right\}$.

## Security pitfalls of Li et al.’s scheme

In this section, we show that Li et al.’s scheme [40] possesses some security vulnerabilities. The following attacks are based on the assumptions that an attacker can extract all of the parameters stored in the smart card by physically monitoring its power consumption [35] and that the attacker can intercept or modify any messages in the public channel [11, 14, 15]. Under these two assumptions, the following problems have been observed and their detailed descriptions are given below.

### Off-line password guessing attack

This attack is an attempt to identify a password until the correct password is found, and is carried out due to the tendency of many users to create simple, brief passwords for the sake of convenience. For this reason, authentication schemes for all password-based users should be designed to prevent a guessing attack; however, Li et al.’s scheme [40] does not provide sufficient protection against such an attack. We therefore propose a scenario for an off-line password-guessing attack using Li et al.’s scheme. The following is a detailed description of this scenario:

Step.1 An attacker extracts {*X*_*u*_, *ID*_*i*_, *h*(⋅), *N*, *s*_1_} from *U*_*i*_’s lost smart card [35].
Step.2 The attacker collects a valid login request message 〈*N*, *ID*_*i*_, *C*_1_, *h*(*r*_1_)〉 from the previous session.
Step.3 The attacker selects a password candidate $PW_{i}^{*}$.
Step.4 The attacker computes *h*(*r*_1_) = *h*(*C*_1_ ⊕ *s*_2_) using *r*_1_ = *C*_1_ ⊕ *s*_2_. Note that *s*_2_ = *h*(*RPW*_*i*_||*s*_1_), and *RPW*_*i*_ = *h*(*X*_*u*_||*PW*_*i*_); therefore, the attacker can write *h*(*r*_1_) = *h*(*C*_1_ ⊕ *h*(*h*(*X*_*u*_||*PW*_*i*_)||*s*_1_)).
Step.5 The attacker then computes $h{\left(r_{1}\right)}^{*}=h(C_{1}⊕h(h(X_{u}||PW_{i}^{*})||s_{1}))$ using the password candidate $PW_{i}^{*}$.
Step.6 The attacker repeats the above steps from 3 to 5 until the computed result *h*(*r*_1_)* is equal to the breached secret *h*(*r*_1_).
Step.7 If *h*(*r*_1_)* and *h*(*r*_1_) correspond, $PW_{i}^{*}$ would be an accurate password. If not, the attacker repeats the above steps until the correct password is found.

Therefore, Li et al’s scheme [40] is vulnerable to the off-line password guessing attack. In addition, if an attacker obtains the user’s password, they can successfully launch a user impersonation attack.

### Server spoofing attack

The security of the password-based user authentication mechanism is based on the intelligence of the password. Thus, if an attacker gains a password, they can pretend to be a legal server. Unfortunately, Li et al.’s scheme allows an attacker to disguise a legal server if the attacker obtains the user’s password *PW*_*i*_ through the guessing attack. The following is a detailed description of this scenario:

Step.1 An attacker extracts {*X*_*u*_, *ID*_*i*_, *h*(⋅), *N*, *s*_1_} from *U*_*i*_’s lost smart card [35].
Step.2 The attacker collects a valid login request message 〈*N*, *ID*_*i*_, *C*_1_, *h*(*r*_1_)〉 from the previous session.
Step.3 The attacker obtains the *PW*_*i*_ through the off-line password guessing attack.
Step.4 The attacker computes *RPW*_*i*_ = *h*(*X*_*u*_||*PW*_*i*_), *s*_2_ = *h*(*RPW*_*i*_||*s*_1_), and *r*_1_ = *s*_2_ ⊕ *C*_1_. The attacker then selects a random number $r_{2}^{*}$ and computes $a^{*}=r_{2}^{*}⊕h(r_{1}\left|\right|s_{2})$ and $b^{*}=h(s_{2}\left|\right|r_{2}^{*}\left|\right|r_{1})$.
Step.5 The attacker then sends an authentication request 〈a*, b*〉 to *U*_*i*_ through a public network.
Step.6 After receiving the 〈a*, b*〉, *U*_*i*_ computes *h*(*r*_1_||*s*_2_), $r_{2}^{\prime }=a^{*}⊕h(r_{1}\left|\right|s_{2})$ and $h(s_{2}||r_{2}^{\prime }||r_{1})$.
Step.7 *U*_*i*_ verifies whether $b^{*}=h(s_{2}\left|\right|r_{2}^{\prime }\left|\right|r_{1})$. Finally, *U*_*i*_ successfully finishes the verification process because b*, which is created by the attacker, is equal to $h(s_{2}||r_{2}^{\prime }||r_{1})$.

Through the aforementioned descriptions, it is demonstrated that the attacker can successfully disguise a legal server in Li et al’s scheme [40].

### Lack of user’s anonymity

In modern networks environments, the leakage of user-related information can expedite an outside attacker to identify every specific user. In such a case, the user’s privacy data is at risk of being disclosed to an untrusted third party who disobeys the user’s will. Therefore, user anonymity is highly considered as a essential property for user authentication mechanism. However, in Li et al.’s scheme [40], an attacker can easily obtain the user’s identity through monitoring the public channels [11, 14, 15] because the user’s *ID*_*i*_ is in plain text form during the login phase. In addition, if the attacker obtains the smart card, the user’s identity *ID*_*i*_ can also be easily exposed through physically monitoring the smart card’s power consumption [35]. With this information, the attacker can also try to launch various types of attacks, which lead to many malicious scenarios. For this reason, user anonymity cannot be preserved in Li et al.’s authentication scheme.

### Absence of password verification process

During the Login phase of Li et al.’s scheme [40], if a user enters his/her identity and password, the smart card does not verify the validity of the password itself. This situation leads to other drawbacks as given below.

Case.1 Assume that the *U*_*i*_ inputs the *ID*_*i*_ and incorrect password $PW_{i}^{*}$ during the login phase; the smart card then computes $RPW_{i}^{*}=h(X_{u}||PW_{i}^{*})$, *h*(*r*_1_), $s_{2}^{*}=h(RPW_{i}^{*}\left|\right|s_{1})$ and $C_{1}^{*}=r_{1}⊕s_{2}^{*}$. *U*_*i*_ then sends a login request $\langle N,ID_{i},C_{1}^{*},h\left(r_{1}\right)\rangle$ to *S*_*j*_ through a public channel. Upon receiving the login request, *S*_*j*_ first checks the validity of the user’s identity *ID*_*i*_. Then, *S*_*j*_ retrieves the stored *R* in the access control table and computes *v* = *h*(*K* ⊕ *ID*_*i*_ ⊕ *R*), $s_{2}^{\prime }=H⊕N⊕v$ and $r_{1}^{\prime }=s_{2}^{\prime }⊕C_{1}^{*}$. *S*_*j*_ then verifies whether $h\left(r_{1}^{\prime }\right)=h\left(r_{1}\right)$. If it holds, *S*_*j*_ accepts the login request. Otherwise, the login request is rejected. However, it is obvious that $h\left(r_{1}^{\prime }\right)\neq h\left(r_{1}\right)$, since $s_{2}^{\prime }$ is not equal to $s_{2}^{*}$. Therefore, *S*_*j*_ belatedly realizes that the entered password $PW_{i}^{*}$ is an incorrect value and *S*_*j*_ rejects *U*_*i*_’s login request. Consequently, if *U*_*i*_ inputs an incorrect password by mistake, the login and verification phases are continued until they have been checked by *S*_*j*_, leading to unnecessary costs in communication and computation, If the password can be quickly verified at the beginning of the login phase, this situation would not occur and the unnecessary waste would be avoided.
Case.2 Assume that an attacker steals a user’s smart card, and logs in to their system using the user’s real *ID*_*i*_ and fake password $PW_{i}^{*}$. The smart card computes a fake login request message $\langle N,ID_{i},C_{1}^{*},h\left(r_{1}\right)\rangle$ with a fake password $PW_{i}^{*}$. If the attacker sends a large number of such fake login request messages, *S*_*j*_ will be busy processing these messages, and will meanwhile reject other legitimate users. Therefore, without a password verification process, the system suffer from a clogging attack, which is a type of denial of service (DoS) attack.

### Inconvenient password change phase

The ideal authentication scheme based on a password should be designed without restricting the user’s ability to alter their password stored in smart cards [11, 15]. Moreover, after carrying out a verification process on an existing password, the self-alteration procedure should be performed within the smart card, without extra communication to server *S*_*j*_, in order to enhance efficiency. However, since a password verification procedure functions with assistance from *S*_*j*_ in Li et al.’s scheme, it is considered to be inefficient and not user-friendly.

### Scalability problem

Li et al.’s scheme [40], in order to strengthen security, it is suggested that the server comprises an access control table to save the information such as the user’s identity, latest login number, and registration times. Accordingly, the server needs to retain each user’s access control table. However, the increased amount of user information needing to be retained places more burden on the server, since the number of access control table will increase as the number of users’ increases. Moreover, the use of the access control table is inefficient in terms of computation time, since the changed values at each phase need to be recorded in the access control table.

## The proposed scheme

**Fig 3:**
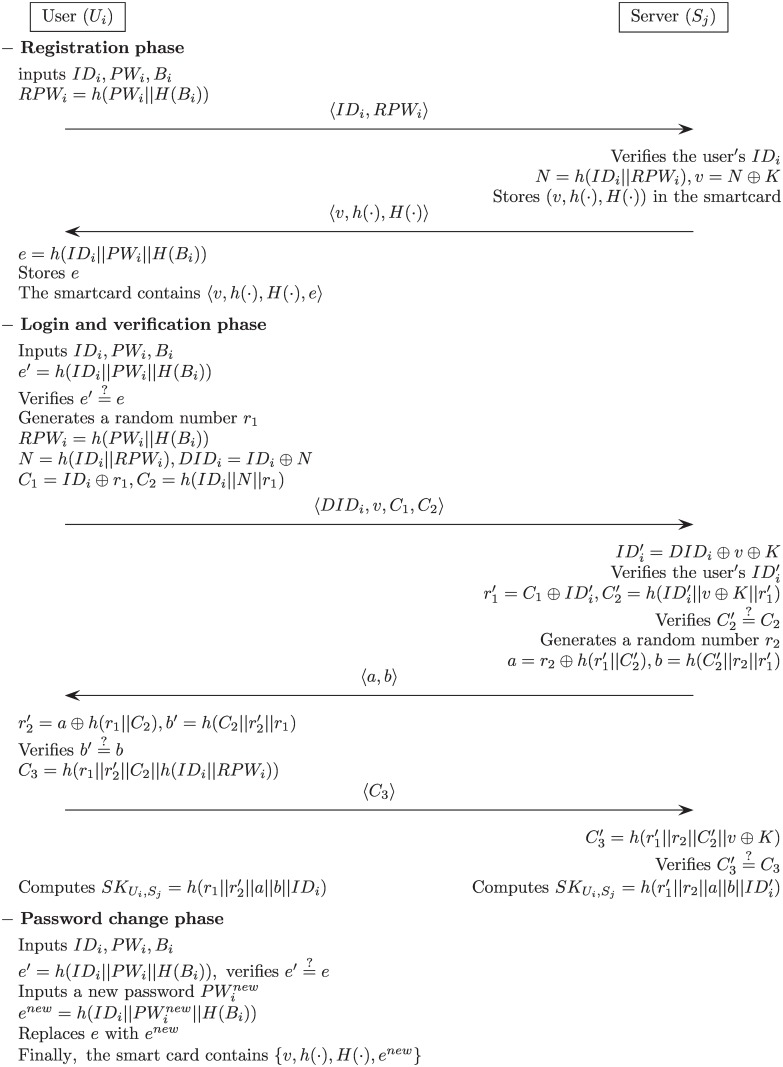
Our proposed scheme.

In this section, we suggest a refined version of the authentication protocol to offer enhanced security by resolving the vulnerabilities of Li et al.’s [40] scheme. In the proposed scheme, in order to resist an off-line password guessing attack and server spoofing attack, we use biometrics information with Biohashing *H*(⋅) [41] to securely conceal the password. We also include a password verification process at the beginning of the login phase to ensure high efficiency and resist a denial of service (DoS) attack. In addition, we remove the access control table to reduce the server load and modify the password change process to provide user-friendly access. Our proposed protocol also consists of four phases: registration, login, verification and password change. Fig 3 describes our proposed scheme, and Table 1 lists the notations employed in the proposed scheme.

### Registration phase

1. *U*_*i*_ inputs *ID*_*i*_ and *PW*_*i*_, and imprints his/her biometrics *B*_*i*_. Then *U*_*i*_ computes *RPW*_*i*_ = *h*(*PW*_*i*_||*H*(*B*_*i*_)) and sends a registration request 〈*ID*_*i*_, *RPW*_*i*_〉 to *S*_*j*_ through a secure channel.
2. *S*_*j*_ verifies the user’s *ID*_*i*_. If it is valid, *S*_*j*_ computes *N* = *h*(*ID*_*i*_||*RPW*_*i*_) and *v* = *N* ⊕ *K*.
3. *S*_*j*_ issues a smart card with the {*v*, *h*(⋅), *H*(⋅)} and sends it to *U*_*i*_ through a secure channel.
4. Upon receiving the smart card, *U*_*i*_ computes *e* = *h*(*ID*_*i*_||*PW*_*i*_||*H*(*B*_*i*_)) and enters it into the smart card’s memory. Finally, the smart card includes the information {*v*, *h*(⋅), *H*(⋅), *e*}.

### Login phase

1. *U*_*i*_ inserts *U*_*i*_’s smart card into a card reader and inputs his/her *ID*_*i*_, *PW*_*i*_, and imprints biometric *B*_*i*_. The smart card then computes *e*′ = *h*(*ID*_*i*_||*PW*_*i*_||*H*(*B*_*i*_)), and compares it with the stored *e* in the smart card. If this holds, the smart card acknowledges the legitimacy of user *U*_*i*_, and proceeds with the next step. Otherwise, it terminates the login process.
2. The smart card selects a random number *r*_1_, and computes *RPW*_*i*_ = *h*(*PW*_*i*_||*H*(*B*_*i*_)), *N* = *h*(*ID*_*i*_||*RPW*_*i*_), *DID*_*i*_ = *ID*_*i*_ ⊕ *N*, *C*_1_ = *ID*_*i*_ ⊕ *r*_1_ and *C*_2_ = *h*(*ID*_*i*_||*N*||*r*_1_).
3. Finally, *U*_*i*_ sends the login request message 〈*DID*_*i*_, *v*, *C*_1_, *C*_2_〉 to *S*_*j*_ through a public network.

### Verification phase

1. *S*_*j*_ computes $ID_{i}^{\prime }=DID_{i}⊕v⊕K$. *S*_*j*_ verifies the user’s $ID_{i}^{\prime }$. If this holds, *S*_*j*_ accepts the login request and proceeds with the next step. Otherwise, *S*_*j*_ rejects the login request and this phase is terminated.
2. *S*_*j*_ computes $r_{1}^{\prime }=C_{1}⊕ID_{i}^{\prime }$, $C_{2}^{\prime }=h(ID_{i}^{\prime }||v⊕K||r_{1}^{\prime })$, and verifies whether $C_{2}^{\prime }=C_{2}$. If this holds, *S*_*j*_ proceeds to the next step. Otherwise, this phase is terminated.
3. *S*_*j*_ selects a random number *r*_2_ and computes $a=r_{2}⊕h(r_{1}^{\prime }\left|\right|C_{2}^{\prime })$ and $b=h(C_{2}^{\prime }||r_{2}||r_{1}^{\prime })$. *S*_*j*_ then sends an authentication request message 〈*a*, *b*〉 to *U*_*i*_ through a public network.
4. *U*_*i*_ computes $r_{2}^{\prime }=a⊕h(r_{1}\left|\right|C_{2})$ and $b^{\prime }=h(C_{2}\left|\right|r_{2}^{\prime }\left|\right|r_{1})$. *U*_*i*_ then verifies whether *b*′ = *b*. If this holds, *U*_*i*_ successfully authenticates *S*_*j*_. Then, *U*_*i*_ computes $C_{3}=h(r_{1}\left|\right|r_{2}^{\prime }\left|\right|C_{2}||h(ID_{i}||RPW_{i}))$ and sends the acknowledgement message 〈*C*_3_〉 to *S*_*j*_ through a public network.
5. *S*_*j*_ computes $C_{3}^{\prime }=h(r_{1}^{\prime }\left|\right|r_{2}\left|\right|C_{2}^{\prime }\left||v⊕K\right)$ and verifies whether $C_{3}^{\prime }=C_{3}$. If this holds, *S*_*j*_ successfully authenticates *U*_*i*_.
6. Finally, *S*_*j*_ computes a shared session key $SK_{U_{i},S_{j}}=h(r_{1}^{\prime }\left|\right|r_{2}||a||b||ID_{i}^{\prime })$ and *U*_*i*_ also computes the same shared session key $SK_{U_{i},S_{j}}=h(r_{1}\left|\right|r_{2}^{\prime }||a||b||ID_{i})$ successfully.

### Password change phase

1. *U*_*i*_ inserts *U*_*i*_’s smart card into a card reader and inputs *ID*_*i*_, and the old password *PW*_*i*_, and then imprints biometric *B*_*i*_. The smart card computes *e*′ = *h*(*ID*_*i*_||*PW*_*i*_||*H*(*B*_*i*_)). The smart card then verifies whether $e_{i}^{\prime }=e_{i}$, where *e*_*i*_ is stored in the smart card. If this condition is not satisfied, it terminates this phase. Otherwise, the smart card proceeds to the next step.
2. *U*_*i*_ inputs a new password $PW_{i}^{new}$, and the smart card then computes $e^{new}=h(ID_{i}||PW_{i}^{new}||H\left(B_{i}\right))$,
3. The smart card replaces the existing value *e* with the new value *e*^*new*^. Finally, the smart card contains the information {*v*, *h*(⋅), *H*(⋅), *e*^*new*^}.

## Security analysis of the proposed scheme

In this section, we first adopt Burrows-Abadi-Needham (BAN) logic [47] to prove that a session key can be correctly generated between *U*_*i*_ and *S*_*j*_. We then verify that our proposed scheme is secure against various attacks through both informal and formal security analysis.

### Authentication proof with BAN logic

In this subsection, based on the BAN logic technique, we verify the legitimacy of session keys distributed to the participants who communicate in our proposed protocol. BAN logic [47] is applied, which is a well-known formal logic technique used to analyze the security of cryptographic protocols. Table 2 shows the basic notations to determine BAN logic.

**Table 2:** Notations.

Notations	Description
*U*∣≡*C*	Condition *C* is believed by *U*
*U*⊲*C*	*U* sees condition *C*
*U*∣∼*C*	*U* expresses the condition *C*
*U* ⇒ *C*	Condition *C* is handled by *U*
♯(*C*)	It makes a fresh *C*
$U\overset{K}{⟷}S$	*U* and *S* share a secret key *K*
<*C*>_*K*_	Combine condition *C* using *K*
(*C*)_*K*_	Perform the hash operation on *C* using *K*

In order to determine the reasonable postulates of BAN logic, five logic rules are also offered as follows.

- Message-meaning rule: $\frac{U|\equiv U\overset{K}{↔}S,U⊲<C{>}_{K}}{U|\equiv S|∼C}$: If *U* trusts that the key *K* is shared with *S*, *U* sees the *C* combined with *K*, then *U* trusts *S* once said *C*.
- Nonce-verification rule: $\frac{U|\equiv \#(C),U|\equiv S|∼C}{U|\equiv S|\equiv C}$: If *U* trusts that *C*’s freshness and *U* trusts *S* once said *C*, then *U* trusts that *S* trusts *C*.
- Believe rule: $\frac{U|\equiv C,U|\equiv M}{A|\equiv (C,M)}$: If *U* trusts *C* and *M*, (*C*, *M*) are also trusted by *U*.
- Freshness-conjuncatenation rule: $\frac{U|\equiv \#(C)}{A|\equiv \#(C,M)}$: If freshness of *C* is trusted by *U*, then *U* can trust the freshness of full condition.
- Jurisdiction rule: $\frac{U|\equiv S|\Rightarrow C,U|\equiv S|\equiv C}{U|\equiv C}$: If *U* trusts that *S* has jurisdiction over *C*, and *U* trusts that *S* trusts a condition *C*, then *U* also trusts *C*.

Through our analysis, we intend to meet the following two goals.

- Goal 1. $U|\equiv (U\overset{sk}{⟷}S)$
- Goal 2. $S|\equiv (U\overset{sk}{⟷}S)$

Next, all transmitted messages in our authentication scheme can be transmuted into an idealized form as follows.

- Message 1. $U\to S:<ID_{i}{>}_{v},<ID_{i}{>}_{r_{1}},{\left(r_{1}\right)}_{ID_{i}}$
- Message 2. $S\to U:<r_{2}{>}_{{\left(r_{1}\right)}_{ID_{i}}},{\left(r_{1},r_{2}\right)}_{ID_{i}}$
- Message 3. *U* → *S*: (*r*_1_, *r*_2_, *ID*_*i*_)_*v*_

In order to analyze our mechanism, we define the following assumptions.

- A1: *U* ∣≡ ♯(*r*_1_)
- A2: *U* ∣≡ ♯(*r*_2_)
- A3: $U|\equiv (U\overset{v}{⟷}S)$
- A4: $S|\equiv (U\overset{v}{⟷}S)$
- A5: $U|\equiv S|\equiv (U\overset{v}{⟷}S)$
- A6: $S|\equiv U|\equiv (U\overset{v}{⟷}S)$

Now, we describe our main proof as follows. In order to describe our proof, we use pre-defined information, including five logic rules, three messages and six assumptions.

According to Message 1, we could derive the following:

- V1: $S⊲<ID_{i}{>}_{v},<ID_{i}{>}_{r_{1}},{\left(r_{1}\right)}_{ID_{i}}$
  Based on assumption A4 and the message meaning rule, we derive:
- V2: *S* ∣≡ *U* ∣∼ *v*
  Based on V2 and the message meaning rule, we derive:
- V3: *S* ∣≡ *U* ∣∼ *ID*_*i*_
  Based on V3 and the message meaning rule, we derive:
- V4: *S* ∣≡ *U* ∣∼ *r*_1_
  Based on assumption A1, V4 and the freshness conjuncatenation rule, we derive:
- V5: $S|\equiv ♯<ID_{i}{>}_{r_{1}}$
  Based on V4, V5 and the nonce verification rule, we derive:
- V6: $S|\equiv U|\equiv <ID_{i}{>}_{r_{1}}$
  Based on V3, V6 and the jurisdiction rule, we derive:
- V7: *S* ∣≡ *r*_1_
  Based on Message 2, we derive:
- V8: $U⊲<r_{2}{>}_{{\left(r_{1}\right)}_{ID_{i}}},{\left(r_{1},r_{2}\right)}_{ID_{i}}$
  Based on assumption A2, V3 and the message meaning rule, we derive:
- V9: *U* ∣≡ *S*∣∼*r*_2_
  Based on assumption A2 and the freshness conjuncatenation rule, we derive:
- V10: $U|\equiv ♯<r_{2}\;{>}_{{\left(r_{1}\right)}_{ID_{i}}}$
  Based on V9, V10 and the nonce verification rule, we derive:
- V11: $U|\equiv S|\equiv \;<r_{2}\;{>}_{{\left(r_{1}\right)}_{ID_{i}}}$
  Based on V9, V11 and the jurisdiction rule, we derive:
- V12: *U* ∣≡ *r*_2_
  Based on *sk*, V12 and assumption A1, we derive:
- V13: $U|\equiv (U\overset{sk}{⟷}S)$ **(Goal 1.)**
  Based on Message 3, we derive:
- V14: *S* ⊲ (*r*_1_, *r*_2_, *ID*_*i*_)_*v*_
  Based on *sk*, V3, V7 and assumption A2, we derive:
- V15: $S|\equiv (U\overset{sk}{⟷}S)$ **(Goal 2.)**

The above description clearly shows that *U*_*i*_ and *S*_*j*_ achieve mutual authentication, and based on (Goal.1) and (Goal.2), *U*_*i*_ and *S*_*j*_ trust that session key *sk* is securely shared between them.

### Informal security analysis

In this subsection, we examine the security our proposed scheme against various attacks, and the suitability of the basic requirements, as described in Section 2.1, are evaluated. Also, we perform a comparative analysis of previous schemes [9, 10, 27, 28, 33, 38, 40], which is illustrated in Table 3. The results are described as follows.

**Table 3:** Security comparison of the proposed scheme and other related schemes.

Features	Hao’s [9]	Jiang’s [10]	Chaudhry’s [27]	Irshad’s [28]	Wu’s [33]	Das’s [38]	Li’s [40]	Proposed Scheme
Proposition 1.	√	√	√	√	×	√	√	√
Proposition 2.	√	√	√	√	×	×	×	√
Proposition 3.	√	√	√	√	√	√	√	√
Proposition 4.	×	×	√	√	×	×	×	√
Proposition 5.	×	×	√	√	×	×	×	√
Proposition 6.	×	×	√	√	×	×	×	√
Proposition 7.	√	√	√	√	√	√	√	√
Proposition 8.	×	×	√	√	×	×	×	√
Proposition 9.	×	√	√	×	×	×	×	√
Proposition 10.	√	√	√	√	√	√	√	√
Proposition 11.	×	×	×	×	√	√	√	√
Proposition 12.	√	√	√	√	√	√	×	√
Formal security proof	×	×	√	√	×	√	√	√

#### Proposition 1. Resistant to insider attack

**Proof.** Insider attack means that an insider can directly obtain the user’s password from the server and can then access the user’s account in another server by using the same password. This attack occurs due to the disclosure of *U*_*i*_’s password *PW*_*i*_ during the registration phase. However, in our scheme, *PW*_*i*_ is transmitted not as a revealed condition but as a form of *RPW*_*i*_ = *h*(*PW*_*i*_||*H*(*B*_*i*_)) with a value of biometrics, *H*(*B*_*i*_), when *U*_*i*_ sends a registration request 〈*ID*_*i*_, *RPW*_*i*_〉 to *S*_*j*_ to prohibit insider attack. Thus, our scheme is secure against insider attack.

#### Proposition 2. Preserve user anonymity

**Proof.** User anonymity is valuable information for the user authentication mechanism, because the disclosure of a user’s identity can allow an unauthorized party to track the user’s login pattern. Our scheme shields user’s identity *ID*_*i*_ transmitted by messages from the potential risks of exposure in order to fulfill user anonymity. Even if an attacker obtains *C*_1_ by snatching login request 〈*DID*_*i*_, *v*, *C*_1_, *C*_2_〉, it is impossible to calculate *ID*_*i*_ since the random number *r*_1_ is not known.

#### Proposition 3. Provide mutual authentication

**Proof.** In the verification phase of our proposed scheme, *U*_*i*_ and *S*_*j*_ can authenticate each other by a number of checking processes. In detail, *S*_*j*_ first verifies the login request by checking whether *C*_2_ is correct. *U*_*i*_ also verifies the authentication request by checking whether *b* is correct. Lastly, *S*_*j*_ verifies the acknowledgement message by checking whether or not *C*_3_ is valid. If all these verification processes are successfully completed, mutual authentication has been executed properly.

#### Proposition 4. Resistant to off-line password guessing attack

**Proof.** An off-line password guessing attack occurs when an attacker attempts to guess a password and eventually finds the exact user’s password in an off-line environment by using the information stored in the smart card or intercepted packets. In our scheme, an attacker can obtain {*v*, *h*(⋅), *H*(⋅), *e*} from the stolen smart card and intercept the login request 〈*DID*_*i*_, *v*, *C*_1_, *C*_2_〉. Using these values, the attacker may try to guess the correct password *PW*_*i*_. However, without knowing *ID*_*i*_ and *H*(*B*_*i*_), the attacker cannot guess *PW*_*i*_. In addition, *H*(*B*_*i*_) is hashed biometric information, which is only known by *U*_*i*_. Therefore, our proposed scheme can withstand an off-line password guessing attack.

#### Proposition 5. Resistant to user impersonation attack

**Proof.** An user impersonation attack occurs when an attacker pretends to be the legal user with the counterfeited login request by using the information that has accumulated from the smart cards and the intercepted packets. Suppose that an attacker obtains {*v*, *h*(⋅), *H*(⋅), *e*} from the stolen smart card. The attacker then generates a random number $r_{1}^{*}$ and attempts to compute *DID*_*i*_ = *ID*_*i*_ ⊕ *N*, $C_{1}^{*}=ID_{i}⊕r_{1}^{*}$ and $C_{2}^{*}=h(ID_{i}\left||N|\right|r_{1}^{*})$. In order to compute *DID*_*i*_, $C_{1}^{*}$, and $C_{2}^{*}$, the attacker must obtain *S*_*j*_’s secret key *K*. However, it is impossible to obtain *K* in our scheme. Therefore, the attacker cannot generate a sufficient login request $\langle DID_{i},v,C_{1}^{*},C_{2}^{*}\rangle$ to deceive *S*_*j*_.

#### Proposition 6. Resistant to server spoofing attack

**Proof.** A server spoofing attack occurs when an attacker masquerades as a legal server with the counterfeited authentication request by using the information stored in the smart card and the intercepted packets. Suppose that an attacker obtains {*v*, *h*(⋅), *H*(⋅), *e*} from the stolen smart card and intercepts the login request 〈*DID*_*i*_, *v*, *C*_1_, *C*_2_〉. The attacker then generates a random number $r_{2}^{*}$ and attempts to modify the authentication request 〈*a*, *b*〉 to impersonate a legal *S*_*j*_. However, the attacker cannot compute a sufficient authentication request to deceive *U*_*i*_ because it is impossible to obtain *r*_1_. In addition, as mentioned above, our scheme can guarantee protection against off-line password guessing attacks. Therefore, our proposed scheme can withstand a sever spoofing attack.

#### Proposition 7. Resistant to replay attack

**Proof.** A replay attack occurs when an attacker deceives a legitimate participant by recycling of the same packets acquired in previous sessions. Suppose that an attacker intercepts the previous login request 〈*DID*_*i*_, *v*, *C*_1_, *C*_2_〉. The attacker might then pretend to be a valid user to login to the server by sending the message. However, if the attacker sends the previous login request, the server would obviously reject the request, because our scheme can detect an invalid random number by comparing it to the *C*_2_ value. In addition, our scheme uses different random numbers in each session. Therefore, our proposed scheme can withstand a replay attack.

#### Proposition 8. Provide a password verification process

**Proof.** It is a possible for a user to input an incorrect password by mistake. However, for the password verification procedure, an incorrect password will be detected after performing the authentication phase. Our scheme overcomes this type of inefficiency problem, evaluating the correctness of the password by verifying the value *e* in the early login phase.

#### Proposition 9. Provide an efficient password change phase

**Proof.** In general, the smart card should carry out the verification process by itself when password alteration occurs. The efficiency of a security scheme can be increased through its own process without communicating to the server *S*_*j*_. Our proposed scheme performs existing password checks in a self-verification process within the smart card. After examining, it will switch the computed value *e*^*new*^ from the new password with the existed value *e* in a convenient and efficient way.

#### Proposition 10. Provide session key agreement

**Proof.** In our scheme, *U*_*i*_ and *S*_*j*_ compute the session key $SK_{U_{i},S_{j}}$ after the mutual authentication process. In addition, the session key is generated by the random number and a one-way hash function. Hence, this session key is different in each session, and it is not possible to derive the session key from the intercepted messages and stolen smart card. Thus, our scheme guarantees that each session key is generated and distributed in a secure way.

#### Proposition 11. No time synchronization

**Proof.** In timestamp-based authentication protocols, when transmitting a message between a user and a server, the clocks of all devices should be synchronized. Therefore, the possibility that an error has occurred is high. However, to avoid this problem, our scheme uses a random numbers *r*_1_, *r*_2_ based authentication mechanism instead of a time-stamp technique.

#### Proposition 12. No access control table

We analyzed the inefficiency risk of Li et al.’s scheme with respect to the access control table. In order to overcome this weakness, our scheme removes the access control table without loss of protection from various types of attacks.

### Formal security analysis

In this subsection, through the formal proof method, we demonstrate that our proposed scheme is secure. First, we specify a hash function as follows.

**Definition 1** A hash function *f*: {0, 1}* → {0, 1}^*n*^ is a one-direction function [48, 49] that takes the input *x* ∈ {0, 1}* of arbitrary length and outputs a bit string with a fixed-length *f*(*x*)∈{0, 1}^*n*^ that is referred to as the “message digest” or “hash value”. When using cryptographic hash functions, the following three common levels of security must be considered:

- It is impossible to acquire input *x* under the conditions of the hash value *y* = *h*(*x*) and the given hash function *h*(⋅).
- It is impossible to acquire other input *x*′, when given the input *x* and *f*(*x*′) = *f*(*x*).
- It is impossible to acquire the inputs (*x*, *x*′), where *x* ≠ *x*′, when given *f*(*x*) = *f*(*x*′).

**Theorem 1** It is assumed that the one-way hash function *h*(⋅) performs like an oracle. Under this assumption, our proposed mechanism is provably secure against an adversary A for protecting the user’s personal information, such as identity *ID*_*i*_, password *PW*_*i*_, biometrics *B*_*i*_, and the server’s secret key *K*.

**Reveal** Given the hash result *y* = *h*(*x*), this random oracle will unconditionally output the input *x*.

**Proof** The similar method used in [20] is applied in our authentication mechanism for verification. In order to prove it, we assume that an attacker A is able to extract the *U*_*i*_’s identity *ID*_*i*_, password *PW*_*i*_, biometrics *B*_*i*_, and the server’s secret key *K*. For this, A runs the experimental algorithm that is shown in Table 4, $EXP_{HASH,A}^{ABUAKAS}$ for our anonymous biometric-based user authentication with key agreement scheme, called ABUAKAS.

**Table 4:** Algorithm $EXP_{HASH,A}^{ABUAKAS}$.

1. Eavesdrop login request message 〈*DID*_*i*_, *v*, *C*_1_, *C*_2_〉
2. Call the Reveal oracle. Let $\left(ID_{i}^{\prime },N^{\prime },r_{1}^{\prime }\right)\leftarrow Reveal\left(C_{2}\right)$
3. Eavesdrop login request message 〈*a*, *b*〉
4. Call the Reveal oracle. Let $\left(C_{2}^{\prime },r_{2}^{\prime },r_{1}^{\prime \prime }\right)\leftarrow Reveal\left(b\right)$
5. Computes $C_{1}^{\prime }=ID_{i}^{\prime }⊕r_{1}^{\prime }$
6. **if $\left(C_{1}^{\prime }=C_{1}\right)$ then**
7. Accepts $ID_{i}^{\prime }$ as the correct *ID*_*i*_ of user
8. Call the Reveal oracle. Let $\left(ID_{i}^{\prime \prime },RPW_{i}^{\prime }\right)\leftarrow Reveal\left(N^{\prime }\right)$
9. **if $\left(ID_{i}^{\prime \prime }=ID_{i}^{\prime }\right)$ then**
10. Call the Reveal oracle. Let $\left(PW_{i}^{\prime },B_{i}^{\prime }\right)\leftarrow Reveal\left(RPW_{i}^{\prime }\right)$
11. Computes $C_{2}^{\prime \prime }=h(ID_{i}^{\prime \prime }\left|\right|N^{\prime }\left|\right|r_{1}^{\prime })$
12. **if $\left(C_{2}^{\prime \prime }=C_{2}^{\prime }\right)$ then**
13. Accept $ID_{i}^{\prime \prime },PW_{i}^{\prime },B_{i}^{\prime }$ as the correct information of *U*_*i*_
*K* as the correct key of *S*_*j*_
14. **return** 1
15. **else**
16. **return** 0
17. **end if**
18. **else**
19. **return** 0
20. **end if**
21. **else**
22. **return** 0
23. **end if**

We define the success probability for $EXP_{HASH,A}^{ABUAKAS}$ as $Success_{HASH,A}^{ABUAKAS}=\left|Pr\left[EXP_{HASH,A}^{ABUAKAS}=1\right]-1\right|$, where $Pr(\dot{)}$ means the probability of $EXP_{HASH,A}^{ABUAKAS}$. The advantage function for this experiment becomes $Adv_{HASH,A}^{ABUAKAS}\left(t,q_{R}\right)=max_{A}$ $Success_{HASH,A}^{ABUAKAS}$ in which the maximum is determined by three factors; all of A, the execution time *t*, and the number of queries *q*_*R*_ derived from Reveal oracle. If the attacker A is assumed that A is able to resolve the hash function problem, A could directly obtain *U*_*i*_’s identity *ID*_*i*_, password *PW*_*i*_, biometrics *B*_*i*_, and the server’s secret key *K*. Refer to attack experiment described in Table 4. In this case, A will discover the complete connections between *U*_*i*_ and *S*_*j*_. However, it is a computationally infeasible problem to invert a one-way hash function *h*(⋅), i.e., $Adv_{HASH,A}^{ABUAKAS}\left(t\right)\leq \in$, ∀*ϵ* > 0. Then, we have $Adv_{HASH,A}^{ABUAKAS}\left(t,q_{R}\right)\leq \in$, since $Adv_{HASH,A}^{ABUAKAS}\left(t,q_{R}\right)$ depends on $Adv_{HASH,A}^{ABUAKAS}\left(t\right)$. Therefore, our proposed scheme is provably secure against the attacker A for deriving *ID*_*i*_, *PW*_*i*_, *B*_*i*_ and *K*.

### Formal security verification using AVISPA tool

In this subsection, we simulate our scheme for the formal security verification using the Automated Validation of Internet Security Protocols and Applications(AVISPA) tool [50] in order to prove that our scheme can withstand both passive and active attacks. We first overview the structure of the AVISPA tool, and then specify our authentication mechanism using High Level Protocols Specification Language(HLPSL) [51]. Lastly, we conduct a simulation using the AVISPA tool and show that our scheme guarantees safety.

#### Overview of AVISPA tool

**Fig 4:**
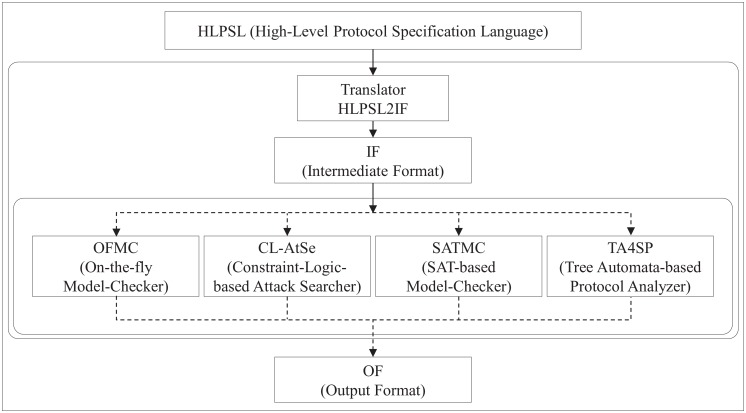
Architecture of AVISPA tool.

AVISPA is a well-known formal method, which has been used to verify the security of protocols. AVISPA provides the specification of security protocols and properties by using a modular and expressive specification language. A number of authentication studies have been conducted [12, 19, 26, 30–32, 39] based on the AVISPA simulation tool. The AVISPA tool can be utilized by external users because the web-interface is accessible from the website [52]. It is also provided as a package(SPAN) that can be installed on the Linux and Mac OS operating systems. The architecture of the AVISPA tool is illustrated in Fig 4.

As shown in Fig 4, the tool takes a specification, as an input, written in HLPSL and produces the results of the test, as an output, from the different back-ends. More specifically, specifications of protocols written in HLPSL are automatically translated by the HLPSL2IF into Intermediate Format (IF) specifications, which are then given as input to the different back-ends. The back-ends consist of four parts [50]: On-the-fly Model-Checker(OFMC), Constraint-Logic-based Attack Searcher(CL-AtSe), SAT based Model Checker(SATMC), and Tree Automata-based on Automatic Approximations for the Analysis of Security Protocols(TA4SP). A detailed description of each part is as follows:

- On-the-fly Model-Checker(OFMC): uses different symbolic techniques to explore the state space in a demand-driven way.
- Constraint-Logic-based Attack Searcher(CL-AtSe): uses simplification heuristics and redundancy elimination techniques.
- SAT based Model Checker(SATMC): uses SAT-solvers in order to find a proposition leading to a fail in the model.
- Tree Automata-based on Automatic Approximations for the Analysis of Security Protocols(TA4SP): uses regular tree languages in order to evaluate the intruder knowledge.

#### Specifying the proposed scheme

**Fig 5:**
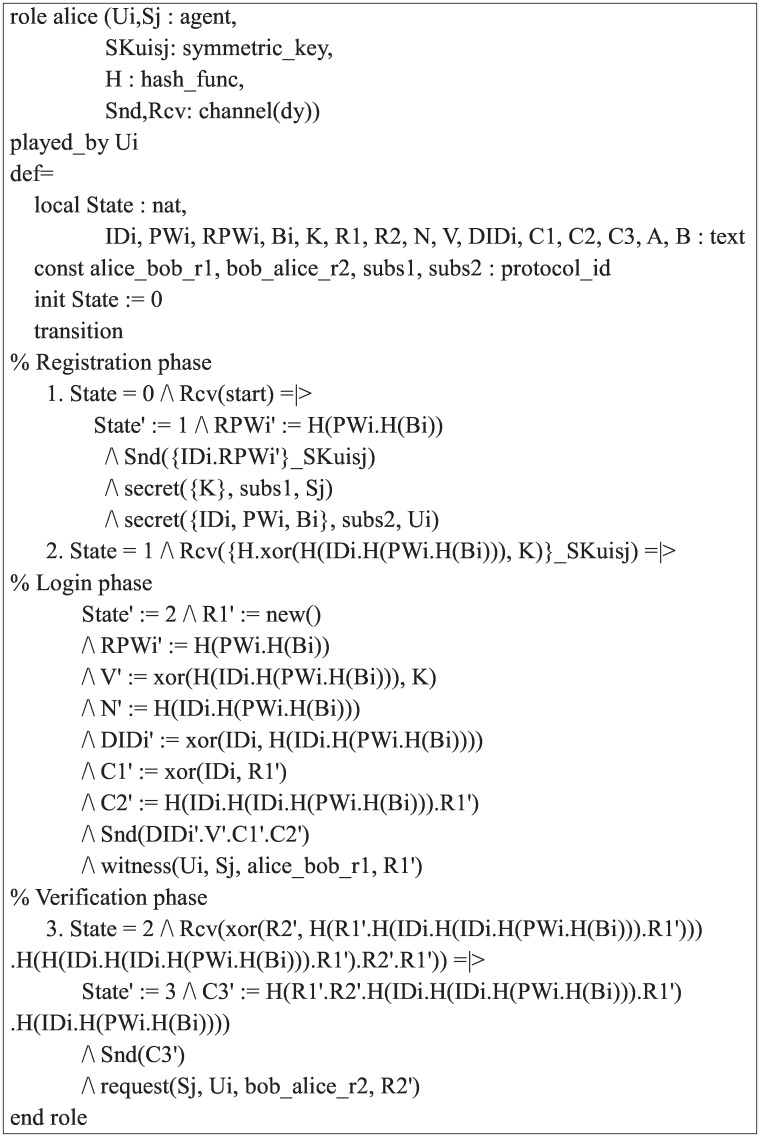
Role specification in HLPSL for the user *U*_*i*_.

This section provides descriptions of the specifications of our scheme in HLPSL. We first implement the basic roles for a user *U*_*i*_ and a server *S*_*j*_ during the registration, login, and verification phase, and then specify the other roles for the session, environment, and goal. In our specifications, the type declaration *channel*(*dy*) indicates that the channel is influenced by the Dolev-Yao threat model [53]. The attacker has full manage over the public channel, such that the attacker can intercept or eavesdrop on all messages sent by agents. In addition, the declaration *secret*({*K*}, *subs*1, *S*_*j*_) indicates that the secret key *K* is only known to *S*_*j*_ and *secret*({*IDi*, *PWi*, *Bi*}, *subs*2, *U*_*i*_) indicates that 〈*ID*_*i*_, *PW*_*i*_, *B*_*i*_〉 are only known to *U*_*i*_. In our implementation, we assume that the bio-hash function *H*(⋅) is the same as the one-way hash function *h*(⋅). The role specification of the user *U*_*i*_ is shown in Fig 5.

**Fig 6:**
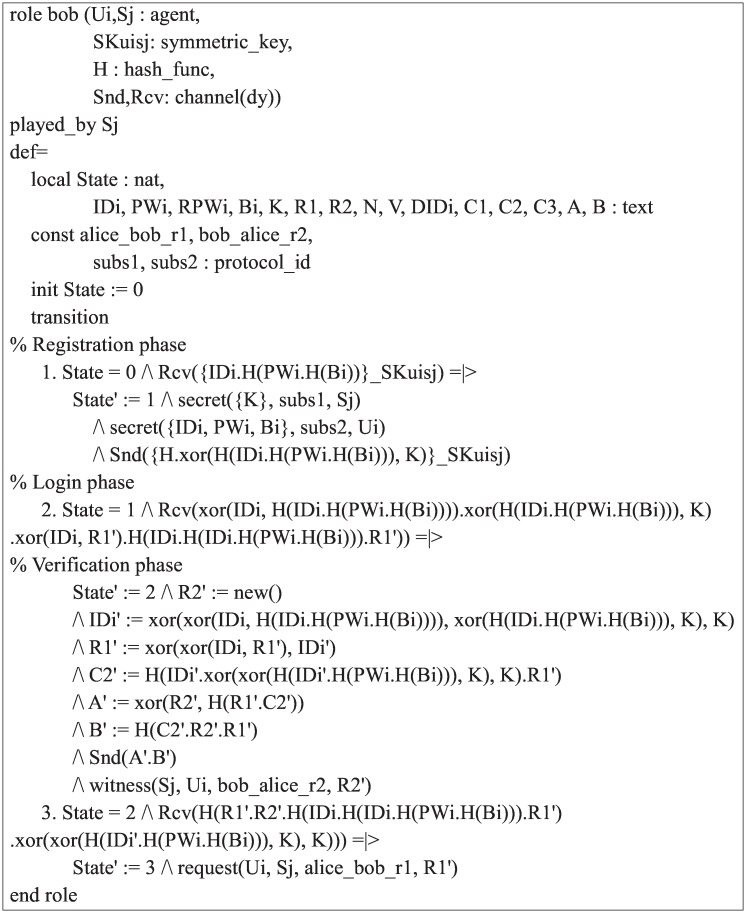
Role specification in HLPSL for the server *S*_*j*_.

During the registration phase, *U*_*i*_ sends a registration request message 〈*ID*_*i*_, *RPW*_*i*_〉 to server *S*_*j*_ through a secure channel using *Snd*() operation, and receives the information {*v*, *h*(⋅), *H*(⋅)} stored in the smart card from *S*_*j*_ using *Rcv*() operation securely. During the login and verification phase, *U*_*i*_ generates a random number *r*_1_ using *new*() operation and sends the login request message 〈*DID*_*i*_, *v*, *C*_1_, *C*_2_〉 to *S*_*j*_ through a public channel. *U*_*i*_ then receives the authentication request message 〈*a*, *b*〉 from *S*_*j*_ through a public channel. Finally, *U*_*i*_ sends the acknowledgement message 〈*C*_3_〉 to *S*_*j*_ through a public channel. The declaration *witness*(*Ui*, *Sj*, *alice*_*bob*_*r*1, *R*1′) indicates that *U*_*i*_ has freshly generated the random number *R*1 for *S*_*j*_. The declaration *request*(*Sj*, *Ui*, *bob*_*alice*_*r*2, *R*2′) denotes that *U*_*i*_ authenticates the server *S*_*j*_. We similarly implemented the role for the server *S*_*j*_ as shown in Fig 6.

During the registration phase, *S*_*j*_ receives the registration request message 〈*ID*_*i*_, *RPW*_*i*_〉 from the user *U*_*i*_ through a secure channel. After receiving the registration request message, the *S*_*j*_ issues a smart card with the {*v*, *h*(⋅), *H*(⋅)} and sends it to *U*_*i*_ through a secure channel using the *Snd*() operation. During the login and verification phase, *S*_*j*_ receives the login request message 〈*DID*_*i*_, *v*, *C*_1_, *C*_2_〉 from the user *U*_*i*_ through a public channel. The *S*_*j*_ then generates a random number *r*_2_ and sends the authentication request message 〈*a*, *b*〉 to *U*_*i*_ through a public channel. Finally, *S*_*j*_ receives the acknowledgement message 〈*C*_3_〉 from the user *U*_*i*_ through a public channel using the *Rcv*() operation. The declaration *witness*(*Sj*, *Ui*, *bob*_*alice*_*r*2, *R*2′) indicates that *S*_*j*_ has freshly generated the random number *R*2 for *U*_*i*_. The declaration *request*(*Ui*, *Sj*, *alice*_*bob*_*r*1, *R*1′) indicates that *S*_*j*_ authenticates the user *U*_*i*_.

**Fig 7:**
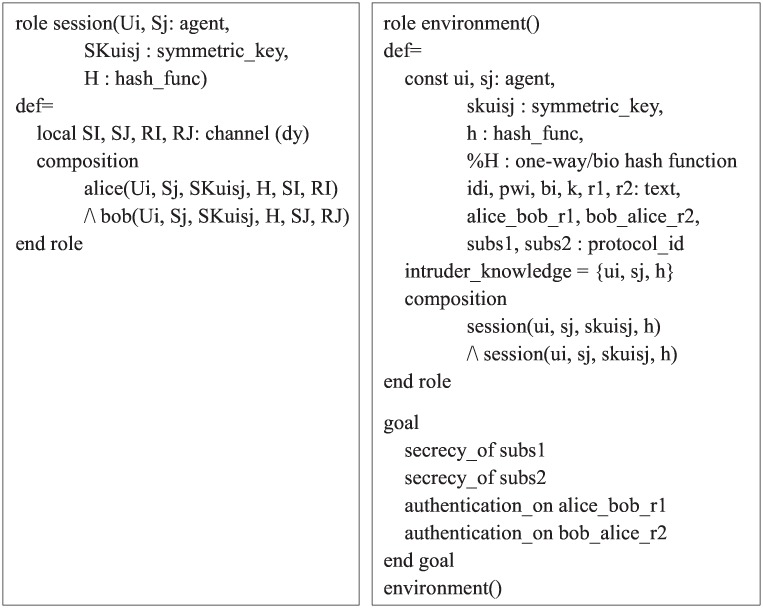
Role specification in HLPSL for the session, environment and goal.

We have therefore provided the specification in HLPSL for the roles including the session, environment, and gaol. The detailed specification of each role is described in Fig 7.

The session part involves the starting parameters, local variables, and composition of agents. The environment part involves the global constants, attacker knowledge, security goals, and the composition of more than one session run in parallel. In our simulation, the following two secrecy goals and two authentications are verified:

- secrecy_of subs1: indicates that the secret key *K* is only known to the legal server *S*_*j*_.
- secrecy_of subs2: indicates that the information including *ID*_*i*_, *PW*_*i*_ and *B*_*i*_ is only known to the legal user *U*_*i*_.
- authentication_on alice_bob_r1: indicates that *U*_*i*_ generates a random number *r*_1_, where *r*_1_ is only taken to *U*_*i*_. If the server *S*_*j*_ securely receives it from the message, *S*_*j*_ then authenticates *U*_*i*_.
- authentication_on bob_alice_r2: indicates that *S*_*j*_ generates a random number *r*_2_, where *r*_2_ is only taken to *S*_*j*_. If the user *U*_*i*_ securely receives it from the message, *U*_*i*_ also authenticates *S*_*j*_.

#### Simulation results

**Fig 8:**
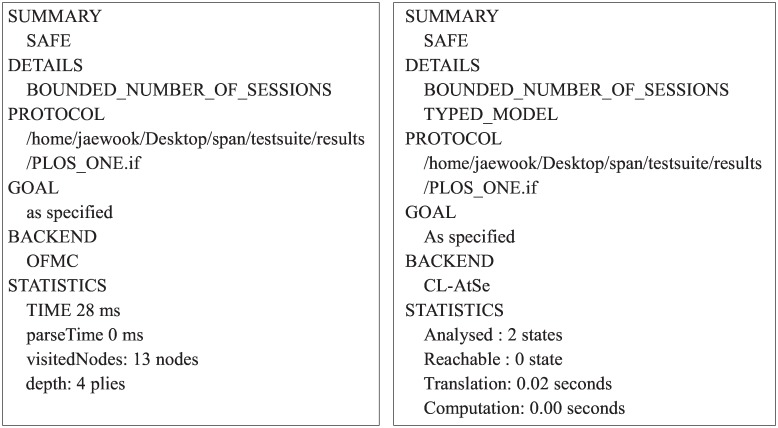
Simulation results under the OFMC and CL-AtSe back-ends.

We simulated our proposed scheme using the AVISPA tool in order to check that our scheme can guarantee safety. The simulation results under the OFMC and CL-AtSe back-ends are shown in Fig 8. The results clearly demonstrate that our scheme is *SAFE* under each bank-end. Therefore, we conclude that our proposed scheme can guarantee protection against passive and active attacks such as replay and man-in-the middle attacks.

## Performance analysis of the proposed scheme

In this section, we have conducted the comparison of the computational costs and execution time for the proposed scheme with other hash-based schemes [34, 38–40]. Generally, the computational cost is examined based on the respective operations in authentication protocol. Accordingly, this analysis of computational cost concentrates on the operations that are conducted by the members, such as user and server. For the evaluation of the computational costs, we define the computational parameter *T*_*H*_ as the time taken to execute a one-way hash function/bio-hash function.

Table 5 provides a summary of the comparison of the computational overheads. Table 5 shows that Lee et al. [34], Das [38], Mir et al. [39], Li et al. [40] and our proposed scheme require the total computational overheads of 19*T*_*H*_, 25*T*_*H*_, 27*T*_*H*_, 26*T*_*H*_, and 21*T*_*H*_, respectively.

**Table 5:** Performance comparison of the proposed scheme and other related schemes.

Phases/Schemes	Lee et al. [34]	Das [38]	Mir et al. [39]	Li et al. [40]	Proposed Scheme
Registration phase	4*T*_*H*_	4*T*_*H*_	5*T*_*H*_	4*T*_*H*_	4*T*_*H*_
Login phase	2*T*_*H*_	3*T*_*H*_	7*T*_*H*_	3*T*_*H*_	5*T*_*H*_
Verification phase	10*T*_*H*_	11*T*_*H*_	10*T*_*H*_	11*T*_*H*_	9*T*_*H*_
Password change phase	3*T*_*H*_	7*T*_*H*_	5*T*_*H*_	8*T*_*H*_	3*T*_*H*_
Total cost	19*T*_*H*_	25*T*_*H*_	27*T*_*H*_	26*T*_*H*_	21*T*_*H*_
Execution time	≈ 3.8ms	≈ 5.0ms	≈ 5.4ms	≈ 5.2ms	≈ 4.2ms

The results show that our proposed scheme is relatively superior to that proposed in a number of related studies [38–40]. In addition, as is shown in Table 3, our proposed scheme guarantees safety against a variety of existing attacks. According to [40], the actual execution times for the one-way hash function *T*_*H*_ is 0.2ms. In Table 5, we also listed the time consumption of our proposed scheme and of the schemes presented in the other related studies [34, 38–40]. Table 5 shows that the execution time of our proposed scheme requires only 4.2 ms (≈ 21 × 0.2 ms); it can therefore be considered as of minor significance. On the other hand, the execution time of Das’s scheme [38], Mir et al.’s scheme [39] and Li et al.’s scheme [40] require 5.0ms (≈ 25 × 0.2 ms), 5.4 ms (≈ 27 × 0.2 ms) and 5.2 ms (≈ 26 × 0.2 ms), respectively; these schemes are therefore proven to be slightly ineffective compared to our scheme. Table 5 demonstrates that our proposed mechanism considers efficiency.

## Conclusions

In this paper, we demonstrate that Li et al.’s scheme has a number of critical vulnerabilities and we propose an extended authentication scheme to overcome these defects. Our proposed scheme has been thoroughly verified in terms of a variety of security features, and the proof result demonstrates that a session key can be correctly generated between the communicating parties. In addition, a performance comparison for the proposed scheme in relation to the schemes proposed in other studies was carried out, and we consider that our proposed scheme has sufficient efficiency and robustness for an integrated EPR information system. In the future, we will propose a new authentication scheme applying the fuzzy extractor technique instead of the biohashing method and analyze the new scheme not only in terms of computation cost, but also in terms of communication and smart card storage cost.
